# Dataset of network simulator related-question posts in stack overflow

**DOI:** 10.1016/j.dib.2022.107942

**Published:** 2022-02-11

**Authors:** Yusuf Sulistyo Nugroho, Syful Islam, Dedi Gunawan, Yogiek Indra Kurniawan, Md. Javed Hossain

**Affiliations:** a Universitas Muhammadiyah Surakarta, Indonesia; b Noakhali Science and Technology University, Bangladesh; c Universitas Jenderal Soedirman, Indonesia

**Keywords:** network simulator, stack overflow, question post, topic modeling

## Abstract

Although the use of network simulator (NS) in predicting the behavior of computer networks has increased, the users often face a variety of challenges and share them on Stack Overflow (SO). However, the challenges that users deal with have not been studied. This paper presents an NS discussion dataset extracted from SOTorrent, which consists of 2,322 NS-related question posts spanning 17 features. The process of data collection was conducted in five steps, including filtering initial post dataset using simulator tags, discovering NS-related tags, collecting the tagged posts, extracting the posts title and preprocessing for LDA (Latent Dirichlet Allocation), and finally applying the LDA topic modeling to obtain the NS posts clustered into eight different topic names. We believe that this dataset will help research community in highlighting issues faced by NS users.

## Specification Table

**Table:** 

Subject	Computer Science
Specific subject area	Networking
Type of data	csv file
How data were acquired	Data were acquired through the largest and most popular question-answering site Stack Overflow (SO).
Data format	Raw: csv file
Parameters for the data collection	To collect the fine grained network simulator posts dataset from SO, we utilize tag as an identifier. We utilize the tag words since these are used to categorize the post to which topic is related to.
Description of the data collection	The data is comprised of all network simulator related question posts (i.e., 2,322 posts) from SO. The dataset has 17 features.
Data Source location	Stack Overflow (SO) Torrent
	URL: https://zenodo.org/record/3255045#.YQFXChMzb0p
Data accessibility	Repository name: **Research Artefact: What network simulator questions do users ask? a large-scale study of stack overflow posts**
	Data Identification Number: 10.5281/zenodo.5118019
	Direct URL to data: https://doi.org/10.5281/zenodo.5118019
Related research article	S. Islam, Y. S. Nugroho, M. J. Hossain, What network simulator questions do users ask? a large-scale study of stack overflow posts, Indonesian Journal of Electrical Engineering and Computer Science 21 (2021) 1622-1633. DOI: http://doi.org/10.11591/ijeecs.v21.i3.pp1622-1633

## Value of the Data

Network simulators (NS) have become a high demand for network engineers and researchers [11]. Different type of users will have various NS-related problems that require a different area of expertise. For example, some users require specific expertise in the tcl scripting, others could have problems on network protocols, or design features. Thus, the difficulties faced by users are likely to differ. The valuable points of our dataset are listed as follows:

• Since users get the benefit from Stack Overflow (SO) to communicate both problems and solutions, this dataset can be useful to understand the most common and pressing NS topics that are frequently faced by the NS community. Besides, identifying the widely discussed NS topics can be an initial step to highlight the topics that are gaining more attention. This is similar to several previous studies that have made used SO posts to identify developers discussion topics relate to Docker [8], IoT [16], Security [17] that are popular in SO.
• This dataset can also help researchers to empirically study the types of questions (i.e., how, what, why) faced by NS users, as same as prior work on mobile-related SO posts [14]. In addition, such analysis will help to identify the nature of difficulties encountered while using NS tools. For example, a prior study investigated the most confusing programming concepts shared in SO by applying a topic modelling analysis [2]. More specific investigation on the trends of NS-related topics in SO can also be performed by using this dataset, similar to a study conducted by Barua et al. [4].
• Researchers may also find our dataset useful to investigate the underlying causes of posting a question in SO. This will help NS community in developing deeper understanding on users information need. For instance, Tian et al. [15] investigated the automatic identification of underlying cause of architecture smell discussions from SO.
• In addition, researchers can also investigate what information (i.e., error message, code etc) is required for a successful question and answer by NS users. For instance, Duijn et al. [7] investigated the information need to post a quality question by developers in SO.

## 1 Data Description

The data presented in this paper is collected and prepared for the purpose of investigating network-simulator-related question posts in Stack Overflow. We used the data to investigate: (i) the types of discussion topics and their popularity, (ii) types of questions that frequently faced by the users, and (iii) the difficulty of topics shared in Stack Overflow.

The raw data was collected from SOTorrent which can be accessed on https://zenodo.org/record/3255045#.YQFXChMzb0p. We subsequently filtered the data based on network-simulator-related tags. The resulted data file accompanying this article consists of 2,322 rows and 17 columns, as presented in Table 1. Every row represents the information of a NS-related question post in Stack Overflow. The properties of each column of the prepared dataset are described in Table 2. Finally, Table 3 shows the topic id and the top 20 keywords suggested by LDA (Latent Dirichlet Allocation) topic modeling on NS dataset.

**Table 1:** Detailed Information of a post.

Name	Description
Id	ID of the post
PostTypeId	Type of the post: 1 represents a question post, and 2 represents an answer post
AcceptedAnswerId	ID of the corresponding accepted answer post for the question post (optional, and appears only when PostTypeId==1)
CreationDate	Creation date of the post
CommunityOwnedDate	Date of the post owned by wiki community
Score	Average score by the viewers for the post
ViewCount	Total number of views for the post (optional, and appears only when PostTypeId==1)
Body	Body of the post
OwnerUserId	ID of the post owner (optional)
Title	Title of the post (optional, and appears only when PostTypeId==1)
Tags	Tags of the post (optional, and appears only when PostTypeId==1)
AnswerCount	Number of answers for the post (optional, and appears only when PostTypeId==1)
CommentCount	Number of comments for the post
FavoriteCount	Number of people who like the post (optional, and appears only when PostTypeId==1)
Dominant_Topic	Topic id predicted by LDA Modeling
Topic_Perc_Contrib	Probability of the topic predicted by LDA modeling
Keywords	List of topic keywords suggested by LDA modeling

**Table 2:** Dataset attributes.

No	Attribute	Data type	Values
1	Id	Numeric	
2	PostTypeId	Numeric	[1, 2]
3	AcceptedAnswerId	Numeric	
4	CreationDate	DateTime	
5	CommunityOwnedDate	DateTime	
6	Score	Numeric	
7	ViewCount	Numeric	
8	Body	String	
9	OwnerUserId	Numeric	
10	Title	String	
11	Tags	String	
12	AnswerCount	Numeric	
13	CommentCount	Numeric	
14	FavoriteCount	Numeric	
15	Dominant_topic	Numeric	[0, 1, 2, 3, 4, 5, 6, 7]
16	Topic_Perc_Contrib	Decimal	Interval: 0–1
17	Keywords	String

**Table 3:** Top 20 keywords suggested by LDA topic modeling for each topics.

Topic_no	Keywords
0	[‘packet’, ‘message’, ‘send’, ‘node’, ‘receive’, ‘application’, ‘multiple’, ‘datum’, ‘specific’, ‘server’, ‘traffic’, ‘layer’, ‘communication’, ‘data’, ‘drop’, ‘rate’, ‘graph’, ‘start’, ‘range’, ‘event’]
1	[‘file’, ‘set’, ‘parameter’, ‘module’, ‘number’, ‘trace’, ‘generate’, ‘script’, ‘random’, ‘position’, ‘access’, ‘give’, ‘connection’, ‘show’, ‘execute’, ‘output’, ‘write’, ‘pass’, ‘modify’, ‘cc’]
2	[‘node’, ‘network’, ‘create’, ‘wireless’, ‘simulator’, ‘simulate’, ‘omnet’, ‘model’, ‘connect’, ‘sensor’, ‘simple’, ‘issue’, ‘address’, ‘interface’, ‘mobile’, ‘host’, ‘system’, ‘resolve’, ‘point’, ‘location’]
3	[‘vein’, ‘omnet’, ‘vehicle’, ‘sumo’, ‘change’, ‘rsu’, ‘car’, ‘scenario’, ‘simulation’, ‘speed’, ‘map’, ‘traci’, ‘current’, ‘model’, ‘flow’, ‘accident’, ‘exist’, ‘makefile’, ‘edge’, ‘tutorial’]
4	[‘simulation’, ‘run’, ‘time’, ‘calculate’, ‘work’, ‘delay’, ‘distance’, ‘end’, ‘throughput’, ‘result’, ‘energy’, ‘base’, ‘measure’, ‘transmission’, ‘power’, ‘channel’, ‘stop’, ‘crash’, ‘record’, ‘ide’]
5	[‘omnet’, ‘inet’, ‘make’, ‘project’, ‘build’, ‘link’, ‘error’, ‘library’, ‘fail’, ‘command’, ‘framework’, ‘import’, ‘version’, ‘define’, ‘user’, ‘store’, ‘check’, ‘input’, ‘runtime’, ‘graph’]
6	[‘error’, ‘omnet’, ‘omnetpp’, ‘variable’, ‘installation’, ‘window’, ‘unable’, ‘read’, ‘problem’, ‘ubuntu’, ‘fix’, ‘compile’, ‘install’, ‘instal’, ‘building’, ‘vector’, ‘package’, ‘implementation’, ‘list’, ‘object’]
7	[‘implement’, ‘find’, ‘add’, ‘function’, ‘route’, ‘code’, ‘class’, ‘type’, ‘program’, ‘call’, ‘source’, ‘protocol’, ‘base’, ‘size’, ‘routing’, ‘method’, ‘path’, ‘miss’, ‘queue’, ‘algorithm’]

## 2 Experimental Design, Materials and Methods

**Fig. 1:**
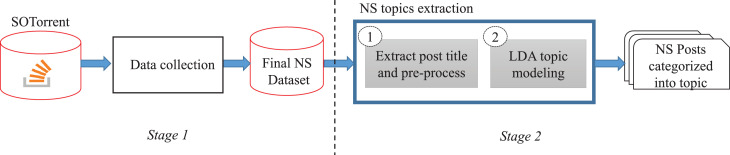
The procedure of the data collection

In the process of preparing the network simulator related dataset presented in [9], we followed the common steps which are also used in the dataset preparation process of similar datasets by [1], [10], [14]. Fig. 1 illustrates the procedures of the data collection which are separated into 2 main stages, that is, (1) raw data collection, and (2) discussion topics extraction using LDA topic modeling.

### 2.1 Data collection

In the first stage, the raw data are collected from the latest version of SO data dump (between July 2008 and December 2019) that is available online on SOTorrent [3]. The initial collected dataset contains 46,947,633 threads with 17 attributes, as shown in Table 2, where it covers 39.83% (18,699,426) question posts and 60.17% (28,248,207) answer posts.

In general, SO question posts contain related tag words aiming to increase the visibility of the posts to other users. To filter the initial NS-related question posts, we utilize the same technique as used in prior case study [1]. The simulator tag is set as the initial tag word which results in 1,407 NS-related question posts. From the resulted posts, the co-occurring tags with simulator are subsequently extracted to discover the NS-related tag words. However, discovering relevant tags have a potential chance to introduce noise in the main dataset. For example, *Set color of node in ns2 with TCL script* is an NS-related post that contains tag words networking together with simulator and ns2. Hence, to mitigate the chance of irrelevant posts in the dataset, we group the target tags through a semi-automatic process. In detail, the validation of the tags is performed by implementing the tag relevance threshold (TRT) and the tag significance threshold (TST).

(1) $$TRT_{tag}=\frac{Number\;of\;NS\;posts\;for\;the\;tag}{Total\;number\;of\;posts\;for\;the\;tag}$$

(2) $$TST_{tag}=\frac{Number\;of\;NS\;posts\;for\;the\;tag}{Number\;of\;NS\;posts\;for\;the\;most\;popular\;tag}$$

where *Number of NS posts for the tag* defines the total number of NS-related question posts for the tag, *Total number of posts for the tag* specifies the total number of posts for the tag, and *Number of NS posts for the most popular tag* describes the number of NS-related posts for the most popular tag. For instance, ns3 is a tag word that co-occurs with simulator tagged post that appears 11 times. Therefore, we also included such tags in the final tag set. This step yields 4 validated tag words, that is simulator, ns2, ns-3, and omnet++, as shown in Table 4. We finally implement these 4 tags to extract 2,322 NS-related question posts from the dataset and used it as the final dataset in the subsequent sections.

**Table 4:** The tags used to identify NS related posts.

Filtered tag	#Initial posts	#Final posts
simulator	1407	1407
ns2	15	599
ns-3	11	316
omnet^++^	11	1406

### 2.2 Topics extraction

In the second stage, the LDA topic modeling is applied to extract the topic from the NS-related threads. First, we perform filtering to remove noisy information from the post titles of the final NS-related dataset by following the same technique as used in previous studies [1], [14]. This pre-processing includes the removal of newline characters, stop words, and emails. We then create the bigram model of the NS-related post title using Gensim^1^ and lemmatize the words to map back in the original words. Finally, to extract the NS topic names, we apply LDA [5], which was also utilized in the prior studies [6], [12], [14], [18]. We adopt the popular Mallet implementation of LDA [13] to group the posts based on the suggested topic and associated keywords in the posts title.

To achieve the number of topics *k* contained in NS-related posts, we run the modeling process and compute the coherence scores for suggested topics number. Here, the coherence score measures the semantic similarity between words in a topic generated by the topic model. The more similar the words in a topic, the higher the coherence score and the better the topic model. In the first step, we run the LDA modeling for the initial range (0-50) with 3 steps increment size. Second, we select the sub-optimal topic range (4-20) based on the LDA computed coherence score. Next, we re-run the model for the sub-optimal range with 1 step increment. This step yields 8 topics (i.e., LDA computed highest coherence score=0.487). Finally, we run the model and obtain 8 NS-related topics with their associated 20 keywords.

## Ethics Statements

Our collected data does not involve human subjects, animal experiments, and social media platforms. We analyzed the data of network-simulator-related question posts from Stack Overflow. Stack Overflow is the most popular question-and-answer website for software developers, providing a large amount of code snippets and free-form text on a wide variety of topics.

## Credit Author Statement

**Yusuf Sulistyo Nugroho** and **Syful Islam:** Conceptualized the study; **Yusuf Sulistyo Nugroho, Syful Islam, Dedi Gunawan, Yogiek Indra Kurniawan** and **Md. Javed Hossain:** Analyzed data and interpreted results; **Yusuf Sulistyo Nugroho, Syful Islam, Dedi Gunawan** and **Yogiek Indra Kurniawan:** Wrote the manuscript; **Dedi Gunawan** and **Md. Javed Hossain** Supervised data collection and overall project.

## Declaration of Competing Interest

The authors declare that they have no known competing financial interests or personal relationships which have, or could be perceived to have, influenced the work reported in this article.

## Footnotes

1 Gensim model: https://radimrehurek.com/gensim/ (Access date: January 2020.)
